# Exploring the perspectives of health care professionals on digital health technologies in pediatric care and rehabilitation

**DOI:** 10.1186/s12984-024-01431-9

**Published:** 2024-09-12

**Authors:** Isabelle Roy, Julia Salles, Erika Neveu, Danaë Lariviére-Bastien, Aurélie Blondin, Danielle Levac, Miriam H. Beauchamp

**Affiliations:** 1 Université de Montréal, Montreal, Canada; 2 CHU Sainte Justine Azrieli Research Center, Montreal, Canada

**Keywords:** Digital health technologies, Pediatric care, Rehabilitation, Healthcare, Virtual reality, Telehealth

## Abstract

**Background:**

Digital health technologies are increasingly used by healthcare professionals working in pediatric hospital and rehabilitation settings. Multiple factors may affect the implementation and use of digital health technologies in these settings. However, such factors have not been identified in a multidisciplinary, pediatric context. The objective of this study was to describe actual use and to identify the factors that promote or hinder the intention to use digital health technologies (mobile learning applications, virtual/augmented reality, serious games, robotic devices, telehealth applications, computerized assessment tools, and wearables) among pediatric healthcare professionals.

**Methods:**

An online survey evaluating opinions, current use, and future intentions to use digital health technologies was completed by 108 professionals at one of Canada’s largest pediatric institutes. Mann-Whitney U tests were used to compare the attitudes of healthcare professionals who intend to increase their use of digital health technologies and those who do not. Linear regression analyses were used to determine predictors of usage success.

**Results:**

Healthcare professionals reported mostly using mobile and tablet learning applications (*n* = 43, 38.1%), telehealth applications (*n* = 49, 43.4%), and computerized assessment tools (*n* = 33, 29.2%). Attitudes promoting the intention to increase the use of digital health technologies varied according to technology type. Healthcare professionals who wished to increase their use of digital health technologies reported a more positive attitude regarding benefits in clinical practice and patient care, but were also more critical of potential negative impacts on patient-professional relationships. Ease of use (*β* = 0.374; *p* = 0.020) was a significant predictor of more favorable usage success. The range of obstacles encountered was also a significant predictor (*β* = 0.342; *p* = 0.032) of less favorable evaluation of usage success. Specific factors that hinder successful usage are lack of training (*β* = 0.303; *p* = 0.033) and inadequate infrastructure (*β* = 0.342; *p* = 0.032).

**Conclusions:**

When working with children, incorporating digital health technologies can be effective for motivation and adherence. However, it is crucial to ensure these tools are implemented properly. The findings of this study underscore the importance of addressing training and infrastructure needs when elaborating technology-specific strategies for multidisciplinary adoption of digital health technologies in pediatric settings.

**Supplementary Information:**

The online version contains supplementary material available at 10.1186/s12984-024-01431-9.

## Background

Digital health technologies are increasingly available to healthcare professionals working in hospital and rehabilitation settings [1–4]. Although there is no consensual definition of digital health technologies, they are typically described as any electronic technology or application that either directly or indirectly supports or provides healthcare, or promotes improved health [5, 6]. Such technologies include, but are not limited to, mobile and tablet learning applications, virtual or augmented reality (VR/AR), serious games, robotic devices, computerized assessment tools, wearables, and mobile health. Mobile health (mHealth) includes virtual care by using telehealth applications for video consultations during telehealth treatment or follow-up.

As digital health development and adoption progresses and access to virtual environments, particularly through VR and serious games, becomes more affordable, interest in using interactive and immersive systems and in exploring the therapeutic value of such systems has grown [7]. This includes exploring the therapeutic potential of such environments alongside other digital health technologies.

### Digital health technologies in healthcare settings

Digital health technologies can assist healthcare professionals for diagnostic, evaluation, treatment, intervention, education, entertainment, and distraction purposes [8, 9]. Several studies have focused on their development and evaluation of their quality and utility (e.g., telehealth applications; [10–12]; mobile learning applications; [13–15]; VR; [16, 17], etc.). Implementation of digital health technologies results in positive outcomes in clinical settings such as fewer hospitalizations, more streamlined tasks, and improved healthcare efficiency and accessibility [18–22]. Additionally, digital health technologies have shown potential in decreasing reliance on specialized equipment and personnel, thereby improving the cost-effectiveness of healthcare services [10, 23, 24]. Specifically, in Canada, the cumulative economic benefits of digital health technologies amounted to 16 billion dollars in savings between 2007 and 2015, primarily through heightened clinician productivity and savings in transportation costs (e.g., with telehealth applications) [23].

### Predicting digital health technology adoption

For digital health technologies to be successfully implemented, it is important to understand the factors that influence the adoption of innovations. The Technology Acceptance Model (TAM; [25]) is a widely used theoretical framework that seeks to explain and predict technology acceptance and usage by individuals. According to this model, users’ behavioral intentions towards technology are primarily determined by two key factors: perceived usefulness and perceived ease of use [26–28].

Perceived usefulness refers to the extent to which individuals believe that using a particular technology will enhance their performance or productivity [29]. Perceived ease of use refers to the degree to which individuals perceive the technology as easy to use [25]. According to this model, these two factors directly influence users’ attitudes and intentions towards adopting and using technology, which in turn predict their actual usage behavior. The TAM has been applied across various domains, including healthcare settings, where it has provided valuable insights into factors influencing the adoption and acceptance of digital health technologies among both healthcare professionals and patients [26, 30, 31].

### Digital health technology needs in pediatric settings

In pediatric settings, digital health technologies present numerous potential advantages over conventional tools. The interactive nature of digital health technologies such as VR, AR and serious games, in particular, enhances motivation and engagement of pediatric patients participating in rehabilitation interventions [32, 33]. Given that children growing up in the current digital age typically have high digital literacy [7], health care systems could benefit from developing innovative digital health applications for care provision, with the goal of improving intervention uptake and compliance, as well as quality and efficiency of care. Digital health technology use in pediatric settings may thus be associated with distinct advantages, necessitating an approach and tailored strategies that consider the specific needs and characteristics of pediatric patients. Likewise, pediatric healthcare professionals may encounter unique challenges related to rapid changes in abilities depending on developmental stages, or family constraints and rules around technology use [34–37].

Multiple factors may affect acceptance and implementation of digital health technologies in pediatric health care settings. In addition, when hospitalized or in rehabilitation, children navigate through multiple services that employ different technologies, suggesting a need to comprehensively study a range of tools and factors at play. While some studies focus on a specific digital health technology such as VR or telemedicine [4, 38], on a specific intent such as improving capacity management in hospitals or improving speech in patients [39–42], or on particular healthcare professions such as occupational therapy or nursing [4, 38, 43], none consider the broader multidisciplinary care context or pediatric population. Previous studies have also reported current usage of digital health technologies, but only a few focus on the intentions behind their adoption [38, 44–46]. This underscores the need for further investigation to better comprehend how to promote the use of digital health technologies.

### Objectives

The objective of this study was to explore the factors that promote and hinder the use of digital health technologies through a survey of multidisciplinary pediatric healthcare professionals. Specific objectives were to provide a description of the use of a variety of digital health technologies in a pediatric hospital and rehabilitation center and; (a) evaluate the attitudes that promote the intention to increase the use of digital health technologies and; (b) identify attitudes associated with, and obstacles that predict, usage success.

The hypotheses were: (a) the intention to increase the use of digital health technologies is associated with more positive attitudes; (b) more positive attitudes predict the best usage success, and conversely, technical problems, lack of training, inadequate infrastructure, time constraints, and other problems predict less usage success.

## Methods

The data were collected as part of a feasibility study regarding the implementation of digital health technologies in pediatric hospitals and rehabilitation centers (the InteRV Project). The project was approved by the local human research ethics committee (CHU Sainte-Justine Azrieli Research Center, reference number 2021–2741). All participants provided informed consent for participation at the beginning of the survey.

### Settings

A survey was completed by healthcare professionals at a Canadian pediatric hospital facility (CHU Sainte-Justine, including the Marie Enfant Rehabilitation Center, Montreal, Canada). CHU Sainte-Justine is one of Canada’s largest pediatric research institutes [47]. The affiliated rehabilitation center provides specialized services in the areas of adaptation-rehabilitation, integration and social participation [48].

### Procedure

Specific services, units, and departments were targeted for survey dissemination to ensure the relevance of the survey for potential participants in terms of likelihood of current or projected use of digital health technologies, and to ensure data generalizability to other pediatric hospitals and rehabilitation centers. Choices were made by the research team in collaboration with clinical collaborators and the hospital directorship to ensure a global perspective. After consultation, the services, units and departments included psychology, nursing, special education, pain management team, psychiatry, speech therapy, physiotherapy, occupational therapy, neurotraumatology, anesthesia and intensive care, and orthopedics.

The survey was initially distributed to managers and service coordinators who shared it directly with their teams via email. It was also promoted in a newsletter for nurses at the hospital, thus broadening its reach within the institution. A total of approximately 1,558 healthcare professionals were reached through these means.

### Participants

Participants (*N* = 108) were 75 healthcare professionals working at the hospital and 33 working at the rehabilitation center. To be included in the study, healthcare professionals had to be working in the targeted services, units and departments. The only exclusion criteria was completion of the survey. The most frequent occupations were nurse (28.7%, *n* = 31), physician (17.6%, *n* = 19), and occupational therapist (9.3%, *n* = 10). Most respondents were women (90.7%, *n* = 97) and were aged between 25 and 34 years (33.3%, *n* = 36) or between 35 and 44 years (32.4%, *n* = 35).

### Measures

The custom-designed survey was built and distributed using the REDCap platform. Study data were also collected and managed using REDCap [49, 50]. The survey focused on the following digital health technologies: mobile and tablet learning applications, virtual or augmented reality, serious games, robotic devices, telehealth applications, computerized assessment tools, and wearables.

Completion time was approximately 10 min. A pilot test of the survey was completed by three research assistants and questions were adjusted based on their feedback. The survey consisted of a total of 20 questions and included information on current digital health technology use, usage intentions and attitudes towards digital health technologies, and obstacles encountered or perceived in relation to using digital health technologies. The nine questions relevant to the current study are in Additional file 1. Four-point scales with anchors on ‘*Success, no obstacles*’ and ‘*Failure, too many obstacles*’ were used for opinions related to usage success. Five-point Likert scales with anchors on ‘*Total agreement*’ and ‘*Total disagreement*’ were used for questions specific to attitudes (Cronbach’s alpha ranging from 0.790 to 0.902).

### Statistical analyses

Analyses were run in IBM SPSS Statistics 29.0. Nonparametric statistics were used for analyzing group differences regarding questions related to attitudes because of the unequal group sizes and the ordinal nature of the data. For each digital health technology, respondents were divided into two groups: healthcare professionals who intend to increase their use of the technology and those who do not. Mann-Whitney U independent samples tests were used to compare the two groups on their attitudes regarding each digital health technology (Additional file 1, questions 8a, 8b, 8c, 8d, 8e, 8f). Due to the large number of tests conducted (6 Mann-Whitney U analyses for each digital health technology), Bonferroni correction was applied to Mann-Whitney U tests; *p*-values smaller than 0.0083 were considered significant.

Parametric statistics were used to investigate predictors of usage success. Two distinct multiple regression models were run. The first regression model explored predictors of usage success among the attitudes and obstacles encountered. The second multiple regression model investigated predictors of usage success among technical problems, lack of training, inadequate infrastructures, lack of time, and other problems. To control for associations with technology experience, both multiple regression models included the variety of digital health technologies used, years of work experience, and the healthcare professional’s job position. Attitudes and usage success questions that were linked to multiple digital health technologies were combined into a single variable by averaging the scales. Statistical significance was assessed at the 0.05 level.

## Results

A total of 154 respondents initiated the survey. Responses from 108 healthcare professionals were included. The remaining 46 respondents were excluded as they only completed the consent form without filling out the survey responses. The response rate (the proportion of respondents who completed the survey out of the total number of individuals in the sample group) ranged between 6.8% and 7.2%. The completion rate (the proportion of respondents who completed the survey out of the number of individuals who initiated it) was 70.1%.

### Experience with digital health technologies

Demographic characteristics of the sample and statistics regarding digital health technology use are presented in Table S1 (Additional file 2). The proportion of healthcare professionals using digital health technologies in their work was 79.6% (*n* = 86), thus 20.4% reported not using any in their practice (*n* = 22). Healthcare professionals reported mostly using mobile and tablet learning applications (*n* = 43, 38.1%), telehealth applications (*n* = 49, 43.4%), and computerized assessment tools (*n* = 33, 29.2%). Some also used VR or AR (*n* = 16, 14.2%), serious games (*n* = 9, 8.0%), robotic devices (*n* = 11, 9.7%), or wearables (*n* = 5, 4.4%). They reported using them mostly for intervention (*n* = 55, 64.0%), evaluation (*n* = 51, 59.3%), and education (*n* = 31, 36.0%) purposes.

### Attitudes promoting the intention to increase the use of digital health technologies

Table 1 presents the values of statistical differences between healthcare professionals who intend to increase their use of digital health technologies and those who do not. Additional file 3 includes Tables S2, S3, S4, S5, S6, S7 and S8 which present the detailed Mann-Whitney U statistics for each digital health technology. Across the majority of digital health technologies, healthcare professionals who plan on increasing their use were also more in agreement with statements 8d and 8e (Additional file 1) regarding the positive impact of digital health technologies and their harmful impact on patient-professional relationship. They were, however, less in agreement with statements 8c and 8f (Additional file 1) concerning the non-essential aspect of digital health technologies, and the limited therapeutic achievement they generate. Only healthcare professionals who intend to increase their use of serious games were more in agreement with statement 8b (Additional file 1) regarding ease of use. No significant results were found for 8a (Additional file 1) concerning the assets of the tools in practice, nor for computerized assessment tools, mobile tablets, and applications for all the questions (8a, 8b, 8c, 8d, 8e, 8f) (Additional file 1).

**Table 1:** Presence of statistical differences between professionals who intend and do not intend to increase their use of digital health technologies

Attitude	Mann-Whitney U values						
	Mobile and tablet learning applications	Virtual or augmented reality	Serious games	Robotic devices	Computerized assessment tools	Telehealth applications	Wearables
Asset in practice	636.50	228.00	175.00	137.50	592.00	553.00	94.00
Ease of use	549.50	180.50	94.00*	80.50	419.50	535.50	69.50
Non-essential	610.00	298.00**	338.50	180.50*	614.00	452.50**	176.50
Positive impact (patient)	739.50	353.50**	264.00**	200.50**	778.00	491.00**	140.00**
Harmful (patient-professional relationship)	526.50	249.50**	267.00*	230.00	534.50	491.50**	155.00
Therapeutic achievement	586.50	185.00**	295.50	232.00	688.50	602.50*	168.00

*p < 0.0083; **p < 0.0017. See Additional file 3 for tables S2, S3, S4, S5, S6, S7 and S8 of detailed Mann-Whitney U statistics for each digital health technology

### Predictors of successful usage

Linear regression results pertaining to attitudes that promote or hinder successful digital health technologies use are presented in Table 2. Ease of use was a significant predictor of more favorable usage success. The range of obstacles encountered (from 0 to 5) was also a significant predictor of less favorable evaluation of usage success. The model demonstrated a moderate fit to the data (adjusted R² = 0.314). The correlation between the observed and predicted values was strong (*R* = 0.671).

**Table 2:** Predictors of successful usage (attitudes)

Predictors	Results		
	Standardized β	*t*	Sig.
Asset in practice	-0.045	-0.282	0.779
Ease of use	0.374	2.425	0.020
Non-essential	0.108	0.771	0.445
Positive impact (patient)	-0.178	-1.006	0.321
Harmful (patient-professional relationship)	0.219	1.647	0.107
Therapeutic achievement	0.121	0.827	0.413
Range of obstacles	0.384	2.571	0.014
Number of digital health technologies used	-0.139	-1.009	0.319
Years of work experience	0.122	0.918	0.364
Job position	-0.125	-0.960	0.343

Linear regression results pertaining to obstacles that promote or hinder successful digital health technologies use are presented in Table 3. Lack of training and inadequate infrastructure were significant predictors of a less favorable evaluation of usage success. The model demonstrated a modest fit to the data (adjusted R²=0.181). The correlation between the observed and predicted values was moderate (*R* = 0.548).

**Table 3:** Predictors of successful usage (obstacles)

Predictors	Results		
	Standardized β	*t*	Sig.
Technical problems	0.249	1.892	0.065
Lack of training	0.303	2.191	0.033
Inadequate infrastructure	0.342	2.207	0.032
Lack of time	-0.024	-0.160	0.874
Other problems	0.184	1.271	0.210
Number of digital health technologies used	-0.184	-1.290	0.203
Years of work experience	0.138	1.042	0.303
Job position	-0.067	-0.525	0.602

For example: difficulties with the therapeutic alliance or delays incurred

## Discussion

The objective of the study was to better understand the attitudes promoting the use of digital health technologies and predictors of their successful usage in a pediatric multidisciplinary setting. The results indicate that the usage of digital health technology is influenced by various attitudes: viewing these technologies as essential, finding them easy to use, recognizing their positive impact on patients, acknowledging the risk of harming the patient-professional relationship, and appreciating their benefits for therapeutic achievement. The main predictor of successful usage was ease of use, and obstacles encountered included lack of training and inadequate infrastructure.

### Attitudes promoting the intention to increase digital health technologies use

As expected, healthcare professionals who plan to increase their use of digital health technologies in their practice generally had a more positive attitude regarding their benefits for patient care compared to those who did not plan to incorporate digital health technologies into their practice. Nonetheless, they viewed the use of digital health technologies as more detrimental to patient-professional relationships and deemed them to be less essential for practice.

These results suggest that professionals who plan on using digital health technologies are aware of the potential drawbacks and acknowledge the obstacles involved in their implementation, but they remain reasonably enthusiastic about future use of these technologies. The fact that they acknowledge that digital health technologies such as mobile/tablet applications, VR or AR, serious games and telehealth applications, may be detrimental to patient-professional relationships could be explained by the considerable amount of equipment that needs to be handled in relation to these tools [51]. This could detract from efficient human interactions, as healthcare professionals may be preoccupied with setting up equipment. Although speculative, healthcare providers in this study appear to demonstrate humility by prioritizing patient benefits over patient-provider relationships and maintaining their therapeutic role. Training could address this issue and offer solutions to preserve both the patient-provider relationship and maximize outcomes [7, 52, 53]. To support this, technical aides or support professionals should be made available to assist with equipment set-up and guide healthcare professionals in the use of digital health technologies.

Furthermore, the current findings indicate that professionals who plan on using digital health technologies view digital health technologies as less essential to the attainment of therapeutic goals. This may be due to the perception that the current treatments are already effective enough to treat patients’ health conditions, and that the addition of digital health technologies may not significantly contribute to treatment outcomes. Previous studies on specific conditions (e.g. diabetes) report digital health technology advantages for patient motivation and entertainment, but not necessarily in terms of their therapeutic value [54]. A review suggests that intervention benefits for some digital health technologies may be more limited for some clinical populations (e.g. Attention Deficit Hyperactivity Disorder (ADHD), Autism Spectrum Disorder (ASD), eating disorders, psychosis, Post Traumatic Stress Disorder) [55]. However, work focusing on robotic devices indicates significant therapeutic value compared to traditional treatments for children living with physical conditions [56]. In line with this disparity, findings suggest that perceptions of therapeutic benefit need to be considered with respect to each individual technology.

Previous work also highlights discrepancies between patient and healthcare professional attitudes toward digital health technologies [57–59]. In general, healthcare professionals and managers exhibit more resistance towards technology compared to patients [43]. Importantly, however, the current findings suggest that professionals’ awareness of digital health technology disadvantages does not diminish their inclination toward their use. One explanation could be that they recognize the potential benefits for children’s motivation and adherence to treatment, which contribute to treatment outcome, and thus disregard the disadvantages.

### Attitudes across digital health technology type

Attitudes promoting the intention to increase the use of digital health technologies varied according to technology type, supporting the need to collect data on individual tools and to elaborate strategies for adopting and implementing specific technologies. For example, healthcare professionals who intend to increase their use of VR/AR, robotic devices, and telehealth applications were more inclined to think that those technologies are non-essential, while there were no significant group differences for other technologies. Professionals may be more guarded in their opinion on potential benefits because of the novelty of those technologies and ongoing changes in their functionalities and applications [60].

Additionally, some digital health technologies such as VR and AR may not be suitable for all patients or medical conditions, and healthcare professionals may need to carefully evaluate each case to determine appropriate treatment options. For example, they may have concerns about the potential risks and side effects of the technology on children, such as cybersickness or disorientation [61]. Moreover, some might be concerned about their ability to use such technology effectively or its appropriateness based on the child’s developmental stage [34, 35].

There are also issues that are unique to pediatric populations who have complex conditions or needs, such as those with ASD or other cognitive or physical conditions. The challenges faced in integrating these technologies with such populations revolve around accessibility, usability, and appropriateness of the technology. For instance, children with ASD may require tailored interfaces or sensory-friendly designs to engage effectively with digital health tools [62], while those with severe physical or intellectual disabilities might have motor deficits or cognitive limitations that hinder their ability to engage and interact with technology [63, 64]. Additionally, concerns regarding data privacy and security are heightened when dealing with vulnerable populations [65].

### Predictors of usage success

The easier healthcare professionals found digital health technologies to use, the more inclined they were to rate their usage success positively, whereas other attitudes were not significant predictors of usage success. A possible explanation for these results could be that healthcare professionals prioritize practical usability when evaluating the success of digital health technologies in their clinical practice, valuing efficiency and convenience in their workflow. This underscores the importance of familiarity and proficiency in using digital health technologies effectively. While this finding has been reported previously [7, 66], this study allows generalization to the multidisciplinary pediatric setting.

As expected, the range of obstacles encountered was also a significant predictor of usage success. The main obstacles to successful use and widespread adoption are inadequate infrastructure and lack of training. These findings align with existing literature, which has shown similar obstacles for a variety of specific technologies. For example, a previous review of facilitators and barriers to VR use in a healthcare setting similarly reported challenges related to environmental context and resources, such as treatment space issues, time to learn how to use, and time to use [7]. The current study extends these observations to a broader spectrum of digital health technologies in the pediatric healthcare setting, which have not been explored to date. Lack of training or lack of familiarity with a new digital health technology logically leads to insufficient skills to use it effectively and could lead to resistance to adopting technology or slow uptake. Appropriate training programs are thus essential to ensure digital health technologies do not have a negative impact, whether perceived or real, on patient care and provider-patient relationships.

The results also underscore the importance of upgrading infrastructures in pediatric settings to ensure healthcare professionals have the resources they need to use technology [67]. Each site may need to assess local infrastructure to ensure that it can accommodate the use of digital health technologies before acquiring new equipment or encouraging professionals to adopt and implement innovative tools.

### Strengths and limitations

The current study encompasses a broad spectrum of disciplines and digital health technologies, offering insights that can be applied across multiple pediatric healthcare centers. Findings can inform decision-makers on effective implementation strategies for these tools and promote the intent to increase their use. Additionally, it enriches conclusions gained from previous studies regarding predictors of usage success, underscoring the need for further changes. Nonetheless, some limitations should be considered. First, the study had a modest sample size and especially small sampling from the rehabilitation center. This may impact generalizability. The response rate for the survey was relatively low, potentially introducing bias and limiting the representativeness of the findings. Low response rate is attributed to the strategy of inviting participation through a weekly, institutional newsletter, which included an audience of approximately 1500 professionals, mostly nurses, which diminishes accuracy and breadth of response [68]. Additionally, 46 participants were excluded due to incomplete surveys, impacting the completion rate. The length of the questionnaire is likely to have contributed to the number of participants aborting the survey prematurely [69] and some respondents may have opened the survey multiple times. Second, a custom-made survey was used. Validated surveys for exploring technology use exist, for example ADOPT-VR [4]; however, a custom survey was chosen to address a broad range of technologies and ensure applicability to the pediatric healthcare setting where the study was conducted. Third, some nonparametric tests were based on unequal sample sizes (e.g. serious games, robotic devices) and this can weaken test accuracy, particularly when one group is much larger. Conclusions regarding these technologies should be interpreted with caution. Finally, other digital health technologies, such as those related to data management for example, were not addressed in this study. Including a wider range of technologies could have encouraged the participation of a greater number of healthcare professionals or managers in the study. Future research with multicentre studies and larger sample sizes should be conducted to further investigate the differences between healthcare professionals working in pediatric hospitals and rehabilitation centers, across various healthcare settings and geographical regions. They should also explore the key factors that contribute to inadequate infrastructure and lack of training and focus on strategies to mitigate such obstacles. Understanding these factors can inform strategies to promote their adoption, ultimately contributing to advancements in pediatric healthcare.

## Conclusion

Considering the attitudes and perceptions of healthcare professionals regarding the integration of digital health technologies into pediatric care will help inform strategies to optimize their implementation and usage. While healthcare professionals intending to increase their use of such technologies generally hold a positive view of their benefits for patient care, they also express concerns about their potential negative impacts on patient-professional relationships and their perceived essentiality for practice. Given the differences between digital health technologies and the main barriers to their use, the findings emphasize the importance of establishing training and implementation tailored specifically to each type of technology and not assuming that the barriers or facilitators generalize across tools. Overall, the successful use of digital health technologies requires a comprehensive approach that carefully considers attitudes, infrastructure, training, and support necessary for their effective implementation and adoption in pediatric care.

## Electronic supplementary material

Below is the link to the electronic supplementary material.

**Additional file 1: Appendix 1:** Survey questions

**Additional file 2: Table S1:** Demographic characteristics and digital health technology use among participants

**Additional file 3: Tables S2–S8**: Comparisons between professionals who intend and do not intend to increase their use of digital health technologies

## Data Availability

The datasets used and/or analysed during the current study are available from the corresponding author on reasonable request.

## Abbreviations

VR: Virtual reality
AR: Augmented reality
