# Supplementary material for: Exploring the perspectives of health care professionals on digital health technologies in pediatric care and rehabilitation
Source: J Neuroeng Rehabil. 2024 Sep 12;21:156. doi: 10.1186/s12984-024-01431-9 (PMC11391714; doi:10.1186/s12984-024-01431-9)
Supplement: Supplementary file 1 — Additional file 1: Appendix 1: Survey questions [file 12984_2024_1431_MOESM1_ESM.docx]

**Additional file 1**

**Appendix 1: Survey questions**

**A – Sociodemographic characteristics**

1. **What position do you hold at CHU Sainte-Justine?**

- Psychologist
- Neuropsychologist
- Special education teacher
- Nurse
- Occupational therapist
- Physiotherapist
- Speech therapist
- Social worker
- Physician
- Other ______
  1. **Which center do you work at?**
     - CHU Sainte-Justine (CHUSJ)
     - Marie-Enfant Rehabilitation Center (CRME)
  2. **Which department do you work in?**
     - Psychology
     - Special Education Team
     - Pain Management Team/"Tout-Doux"
     - Psychiatry
     - Speech Therapy
     - Physiotherapy
     - Occupational Therapy
     - Neurotraumatology
     - Anesthesia-Resuscitation
     - Orthopedics Clinic
     - CRME: Deficiency and Neurotrauma
     - Other _____

1. **How long have you been in this position?**
   - Less than 5 years
   - Between 5 and 10 years
   - Between 10 and 15 years
   - Between 15 and 20 years
   - More than 20 years
2. **What is your gender identity?**
   - Woman
   - Man
   - I identify as ______
   - I would rather not answer
3. **What age range are you in?**
   - 18 to 24 years old
   - 25 to 34 years old
   - 35 to 44 years old
   - 45 to 54 years old
   - 55 to 64 years old
   - 65 years old and more
   - I would rather not answer

**B – Digital health technology use**

1. **Have you ever used a digital health technology in your clinical practice at CHU Sainte-Justine or the Marie-Enfant Rehabilitation Center?**

- Mobile and tablet learning applications
- Virtual or augmented reality
- Serious games
- Robotic devices
- Computerized assessment tools
- Telemedicine/teletherapy applications
- Wearables
- Other (specify) ______
- I have not used any digital health technology

**C - Usage success of digital health technologies**

1. **For each digital health technology mentioned below, how would you describe your experience?**

Mobile and tablet learning applications

Virtual or augmented reality

Serious games

Robotic devices

Computerized assessment tools

Telemedicine/teletherapy applications

Wearables

- - 1- The use of digital health technologies was successful, no obstacles or problems occurred.
  - 2- Some minor obstacles or problems occurred, but the use was successful.
  - 3- Several obstacles or problems hindered my use of the digital health technologies.
  - 4- The use of technological tools was a failure, too many obstacles or problems occurred.
  - 5- Not applicable (NA)

1. **What general obstacles did you encounter when using technological tools?**

- Technical problems
- Lack of training
- Inadequate facilities
- Lack of time (e.g. to familiarize with the digital health technology)
- Other problems

**D - Attitudes regarding digital health technologies**

1. **For each digital health technology mentioned below, to what extent do you agree with the following statements?**

Mobile and tablet learning applications

Virtual or augmented reality

Serious games

Robotic devices

Computerized assessment tools

Telemedicine/teletherapy applications

Wearables

1. **I think it is an asset in my clinical practice** (Asset in practice)
   - 1- Total agreement
   - 2- Agree
   - 3- Somewhat agree
   - 4- Disagree
   - 5- Total disagreement
   - 0- I don’t know
2. **I find it easy to use** (Ease of use)
   - 1- Total agreement
   - 2- Agree
   - 3- Somewhat agree
   - 4- Disagree
   - 5- Total disagreement
   - 0- I don’t know
3. **I don’t think it adds anything to my clinical practice** (Non-essential)
   - 1- Total agreement
   - 2- Agree
   - 3- Somewhat agree
   - 4- Disagree
   - 5- Total disagreement
   - 0- I don’t know
4. **I think it can have a positive impact on the patient and their care** (Positive impact (patient))
   - 1- Total agreement
   - 2- Agree
   - 3- Somewhat agree
   - 4- Disagree
   - 5- Total disagreement
   - 0- I don’t know
5. **I think it harms the quality of the patient-professional relationship** (Harmful (patient-professional relationship))
   - 1- Total agreement
   - 2- Agree
   - 3- Somewhat agree
   - 4- Disagree
   - 5- Total disagreement
   - 0- I don’t know
6. **It allows for therapeutic goals that would be unattainable without it** (Therapeutic achievement)
   - 1- Total agreement
   - 2- Agree
   - 3- Somewhat agree
   - 4- Disagree
   - 5- Total disagreement
   - 0- I don’t know
7. **I intend to increase my use of the following digital health technologies:**

- Mobile and tablet learning applications
- Virtual or augmented reality
- Serious games
- Robotic devices
- Computerized assessment tools
- Telemedicine/teletherapy applications
- Wearables
