# Supplementary material for: Exploring the perspectives of health care professionals on digital health technologies in pediatric care and rehabilitation
Source: J Neuroeng Rehabil. 2024 Sep 12;21:156. doi: 10.1186/s12984-024-01431-9 (PMC11391714; doi:10.1186/s12984-024-01431-9)
Supplement: Supplementary file 2 — Additional file 2: Table S1: Demographic characteristics and digital health technology use among participants [file 12984_2024_1431_MOESM2_ESM.docx]

**Additional file 2**

**Table S1 : Demographic characteristics and digital health technology use among participants**

|  | | n (%) |
| --- | --- | --- |
| **Job position** | Psychologist and neuropsychologist | 15 (13.9) |
|  | Special educator, social worker, psychoeducator | 15 (13.9) |
|  | Occupational therapist, physiotherapist, orthotist | 17 (15.7) |
|  | Physician | 19 (17.6) |
|  | Nurse | 31 (28.7) |
|  | Other | 11 (10.2) |
| **Center** | Hospital | 75 (69.4) |
|  | Rehabilitation Center | 33 (30.6) |
| **Gender identity** | Woman | 97 (90.7) |
|  | Man | 10 (9.3) |
| **Age range** | 18 to 24 years old | 6 (5.6) |
|  | 25 to 34 years old | 36 (33.3) |
|  | 35 to 44 years old | 35 (32.4) |
|  | 45 to 54 years old | 20 (18.5) |
|  | 55 to 65 years old | 11 (10.2) |
| **Job experience** | Less than 5 years | 41 (38.0) |
|  | Between 5 and 10 years | 20 (18.5) |
|  | Between 10 and 15 years | 16 (14.8) |
|  | Between 15 and 20 years | 10 (9.3) |
|  | More than 20 years | 21 (19.4) |
| **Digital health technologies used** | Mobile and tablet learning applications | 43 (39.8) |
|  | Virtual or augmented reality | 16 (14.8) |
|  | Serious games | 9 (8.3) |
|  | Robotic devices | 11 (10.2) |
|  | Computerized assessment tools | 33 (30.6) |
|  | Telemedicine/teletherapy applications | 49 (45.4) |
|  | Wearables | 5 (4.6) |
|  | Others | 15 (13.9) |
| **Digital health technologies desired** | Mobile and tablet learning applications | 54 (50.0) |
|  | Virtual or augmented reality | 37 (34.3) |
|  | Serious games | 27 (25.0) |
|  | Robotic devices | 21 (19.4) |
|  | Computerized assessment tools | 58 (53.7) |
|  | Telemedicine/teletherapy applications | 42 (38.9) |
|  | Wearables | 25 (23.1) |
|  | Others | 6 (5.6) |
