# Supplementary material for: Exploring the perspectives of health care professionals on digital health technologies in pediatric care and rehabilitation
Source: J Neuroeng Rehabil. 2024 Sep 12;21:156. doi: 10.1186/s12984-024-01431-9 (PMC11391714; doi:10.1186/s12984-024-01431-9)
Supplement: Supplementary file 3 — Additional file 3: Tables S2–S8: Comparisons between professionals who intend and do not intend to increase their use of digital health technologies [file 12984_2024_1431_MOESM3_ESM.docx]

**Additional file 3**

Tables S2-S8 : Comparisons between professionals who intend and do not intend to increase their use of digital health technologies

| **Table S2: Comparisons between professionals who intend and do not intend to increase their use of mobile and tablet applications** | | | | | | | |
| --- | --- | --- | --- | --- | --- | --- | --- |
| **Attitudes (items ranked on a 5-point scale)** | **High Adopters** | | **Low or Neutral Adopters** | | **Z** | **U** | **Asymp. Sig. (2-tailed)** |
|  | n | Mean Rank | n | Mean Rank |  |  |  |
| **Asset in practice** | 47 | 37.54 | 29 | 40.05 | -0.540 | 636.500 | 0.589 |
| **Ease of use** | 44 | 34.99 | 26 | 36.37 | -0.293 | 549.500 | 0.770 |
| **Non-essential** | 48 | 46.79 | 35 | 35.43 | -2.322 | 610.000 | 0.020 |
| **Positive impact (patient)** | 53 | 40.95 | 37 | 52.01 | -2.225 | 739.500 | 0.026 |
| **Harmful (patient-professional relationship)** | 47 | 35.20 | 33 | 48.05 | -2.543 | 526.500 | 0.011 |
| **Therapeutic achievement** | 47 | 42.52 | 31 | 34.92 | -1.572 | 586.500 | 0.116 |
| **p<0.0083** | | | | | | | |

| **Table S3: Comparisons between professionals who intend and do not intend to increase their use of virtual/augmented reality** | | | | | | | |
| --- | --- | --- | --- | --- | --- | --- | --- |
| **Attitudes (items ranked on a 5-point scale)** | **High Adopters** | | **Low or Neutral Adopters** | | **Z** | **U** | **Asymp. Sig. (2-tailed)** |
|  | n | Mean Rank | n | Mean Rank |  |  |  |
| **Asset in practice** | 24 | 22.00 | 31 | 32.65 | -2.579 | 228.000 | 0.010 |
| **Ease of use** | 23 | 19.85 | 19 | 23.50 | -1.024 | 180.500 | 0.306 |
| **Non-essential** | 29 | 47.72 | 43 | 28.93 | -3.874 | 298.000 | **<0.001** |
| **Positive impact (patient)** | 31 | 27.40 | 46 | 46.82 | -4.014 | 353.500 | **<0.001** |
| **Harmful (patient-professional relationship)** | 28 | 23.41 | 33 | 37.44 | -3.186 | 249.500 | **0.001** |
| **Therapeutic achievement** | 27 | 41.15 | 34 | 22.94 | -4.294 | 185.000 | **<0.001** |
| **p<0.0083** | | | | | | | |

| **Table S4: Comparisons between professionals who intend and do not intend to increase their use of serious game** | | | | | | | |
| --- | --- | --- | --- | --- | --- | --- | --- |
| **Attitudes (items ranked on a 5-point scale)** | **High Adopters** | | **Low or Neutral Adopters** | | **Z** | **U** | **Asymp. Sig. (2-tailed)** |
|  | n | Mean Rank | n | Mean Rank |  |  |  |
| **Asset in practice** | 16 | 19.44 | 35 | 29.00 | -2.259 | 175.000 | 0.024 |
| **Ease of use** | 17 | 14.53 | 22 | 24.23 | -2.785 | 94.000 | **0.005** |
| **Non-essential** | 23 | 41.28 | 44 | 30.19 | -2.269 | 338.500 | 0.023 |
| **Positive impact (patient)** | 24 | 23.50 | 51 | 44.82 | -4.164 | 264.000 | **<0.001** |
| **Harmful (patient-professional relationship)** | 22 | 23.66 | 41 | 36.48 | -2.727 | 267.000 | **0.006** |
| **Therapeutic achievement** | 22 | 41.07 | 43 | 28.87 | -2.597 | 295.500 | 0.009 |
| **p<0.0083** | | | | | | | |

| **Table S5: Comparisons between professionals who intend and do not intend to increase their use of robotic devices** | | | | | | | |
| --- | --- | --- | --- | --- | --- | --- | --- |
| **Attitudes (items ranked on a 5-point scale)** | **High Adopters** | | **Low or Neutral Adopters** | | **Z** | **U** | **Asymp. Sig. (2-tailed)** |
|  | n | Mean Rank | n | Mean Rank |  |  |  |
| **Asset in practice** | 14 | 17.32 | 28 | 23.59 | -1.686 | 137.500 | 0.092 |
| **Ease of use** | 13 | 13.19 | 19 | 18.76 | -1.759 | 80.500 | 0.079 |
| **Non-essential** | 17 | 41.38 | 43 | 26.20 | -3.111 | 180.500 | **0.002** |
| **Positive impact (patient)** | 18 | 20.64 | 45 | 36.54 | -3.254 | 200.500 | **0.001** |
| **Harmful (patient-professional relationship)** | 19 | 22.11 | 37 | 31.78 | -2.191 | 230.000 | 0.028 |
| **Therapeutic achievement** | 16 | 34.00 | 40 | 26.30 | -1.710 | 232.000 | 0.087 |
| **p<0.0083** | | | | | | | |

| **Table S6: Comparisons between professionals who intend and do not intend to increase their use of computerized assessment tools** | | | | | | | |
| --- | --- | --- | --- | --- | --- | --- | --- |
| **Attitudes (items ranked on a 5-point scale)** | **High Adopters** | | **Low or Neutral Adopters** | | **Z** | **U** | **Asymp. Sig. (2-tailed)** |
|  | n | Mean Rank | n | Mean Rank |  |  |  |
| **Asset in practice** | 45 | 37.84 | 28 | 35.64 | -0.478 | 592.000 | 0.633 |
| **Ease of use** | 40 | 30.99 | 23 | 33.76 | -0.638 | 419.500 | 0.523 |
| **Non-essential** | 48 | 41.71 | 30 | 35.97 | -1.179 | 614.000 | 0.239 |
| **Positive impact (patient)** | 52 | 41.46 | 36 | 48.89 | -1.490 | 778.000 | 0.136 |
| **Harmful (patient-professional relationship)** | 46 | 35.12 | 30 | 43.68 | -1.717 | 534.500 | 0.086 |
| **Therapeutic achievement** | 47 | 38.65 | 30 | 39.55 | -0.185 | 688.500 | 0.853 |
| **p<0.0083** | | | | | | | |

| **Table S7: Comparisons between professionals who intend and do not intend to increase their use of telehealth** | | | | | | | |
| --- | --- | --- | --- | --- | --- | --- | --- |
| **Attitudes (items ranked on a 5-point scale)** | **High Adopters** | | **Low or Neutral Adopters** | | **Z** | **U** | **Asymp. Sig. (2-tailed)** |
|  | n | Mean Rank | n | Mean Rank |  |  |  |
| **Asset in practice** | 39 | 34.18 | 40 | 45.68 | -2.372 | 553.000 | 0.018 |
| **Ease of use** | 37 | 33.47 | 35 | 39.70 | -1.345 | 535.500 | 0.179 |
| **Non-essential** | 38 | 55.59 | 48 | 33.93 | -4.246 | 452.500 | **<0.001** |
| **Positive impact (patient)** | 40 | 32.78 | 51 | 56.37 | -4.554 | 491.000 | **<0.001** |
| **Harmful (patient-professional relationship)** | 39 | 32.60 | 45 | 51.08 | -3.584 | 491.500 | **<0.001** |
| **Therapeutic achievement** | 37 | 53.72 | 51 | 37.81 | -2.977 | 602.500 | **0.003** |
| **p<0.0083** | | | | | | | |

| **Table S8: Comparisons between professionals who intend and do not intend to increase their use of wearables** | | | | | | | |
| --- | --- | --- | --- | --- | --- | --- | --- |
| **Attitudes (items ranked on a 5-point scale)** | **High Adopters** | | **Low or Neutral Adopters** | | **Z** | **U** | **Asymp. Sig. (2-tailed)** |
|  | n | Mean Rank | n | Mean Rank |  |  |  |
| **Asset in practice** | 15 | 14.27 | 21 | 21.52 | -2.180 | 94.000 | 0.029 |
| **Ease of use** | 13 | 12.35 | 14 | 15.54 | -1.165 | 69.500 | 0.244 |
| **Non-essential** | 22 | 26.48 | 23 | 19.67 | -1.816 | 176.500 | 0.069 |
| **Positive impact (patient)** | 20 | 17.50 | 29 | 30.17 | -3.322 | 140.000 | **<0.001** |
| **Harmful (patient-professional relationship)** | 19 | 18.16 | 21 | 22.62 | -1.261 | 155.000 | 0.207 |
| **Therapeutic achievement** | 19 | 27.16 | 26 | 19.96 | -2.019 | 168.000 | 0.043 |
| **p<0.0083** | | | | | | | |
